# Comparative analysis of the quality of a global algorithm and a local algorithm for alignment of two sequences

**DOI:** 10.1186/1748-7188-6-25

**Published:** 2011-10-27

**Authors:** Valery O Polyanovsky, Mikhail A Roytberg, Vladimir G Tumanyan

**Affiliations:** 1 Engelhardt Institute of Molecular Biology, RAS, 119991, Moscow, Russia; 2 Institute of Mathematical Problems in Biology, RAS, 142290, Pushchino, Russia

## Abstract

**Background:**

Algorithms of sequence alignment are the key instruments for computer-assisted studies of biopolymers. Obviously, it is important to take into account the "quality" of the obtained alignments, i.e. how closely the algorithms manage to restore the "gold standard" alignment (GS-alignment), which superimposes positions originating from the same position in the common ancestor of the compared sequences. As an approximation of the GS-alignment, a 3D-alignment is commonly used not quite reasonably. Among the currently used algorithms of a pair-wise alignment, the best quality is achieved by using the algorithm of optimal alignment based on affine penalties for deletions (the Smith-Waterman algorithm). Nevertheless, the expedience of using local or global versions of the algorithm has not been studied.

**Results:**

Using model series of amino acid sequence pairs, we studied the relative "quality" of results produced by local and global alignments versus (1) the relative length of similar parts of the sequences (their "cores") and their nonhomologous parts, and (2) relative positions of the core regions in the compared sequences. We obtained numerical values of the average quality (measured as accuracy and confidence) of the global alignment method and the local alignment method for evolutionary distances between homologous sequence parts from 30 to 240 PAM and for the core length making from 10% to 70% of the total length of the sequences for all possible positions of homologous sequence parts relative to the centers of the sequences.

**Conclusion:**

We revealed criteria allowing to specify conditions of preferred applicability for the local and the global alignment algorithms depending on positions and relative lengths of the cores and nonhomologous parts of the sequences to be aligned. It was demonstrated that when the core part of one sequence was positioned above the core of the other sequence, the global algorithm was more stable at longer evolutionary distances and larger nonhomologous parts than the local algorithm. On the contrary, when the cores were positioned asymmetrically, the local algorithm was more stable at longer evolutionary distances and larger nonhomologous parts than the global algorithm. This opens a possibility for creation of a combined method allowing generation of more accurate alignments.

## 1. Background

Pair-wise alignment of amino acid sequences is the main method of comparative protein analysis. Among the most popular algorithms based on comparison of protein primary structures the Needleman-Wunch algorithm [1], the Smith-Waterman algorithm [2], BLAST [3], and FASTA [4] should be noted. On the basis of paper [1] the algorithm [5] was created for comparing sequences with intermittent similarities. The improved version [6] makes use of multiple parameter sets in computation of an optimal alignment of the two sequences. A number of algorithms (Walquist *et al*. [7], Litvinov *et al*. [8], etc.) also take into account specific features of protein primary structures. However, it is important to know how closely algorithmic alignments produced through optimization of any chosen target function reflect an evolution-based alignment of the appropriate amino acid sequences, e.g. the one, which juxtaposes the positions in the compared proteins originating from the same position in their common predecessor.

The "quality" of the alignment algorithms, i.e. mutual concordance of algorithmic and GS alignments, was analyzed from different points of view; in most cases, alignments based on intercomparison of three-dimensional structures were used as the GS alignments. It was premised on the fact that 3D structures of proteins are much more conservative than their amino acid sequences [9].

In other words, sequences corresponding to a certain fold are greatly confluent: the same structure corresponds to somewhat dissimilar or even totally dissimilar sequences. There are also a number of counter-examples, when similar sequences correspond to totally different 3D structures, but such examples are much less common [10]. Vingron and Argos [11] demonstrated that there was a relationship between conservatism of the optimal global alignment region in a set of suboptimal alignments and its similarity with the structural alignment results. They showed that regions of optimal alignment, recurring most frequently in suboptimal alignments, were very similar to alignments produced by the structural alignment methods.

In works [12,13], evaluation of the accuracy of the optimal alignment was based on determination of the matching accuracy for each pair of matched amino acid residues, with the following plotting of the robustness index values versus the number of the aligned pair of residues. For example, Mevissen and Vingron [12] used a weight difference for the optimal alignment and the alignment with the largest weight, in which residue *i *and residue *j *were not matched, as a measure of robustness for matching residue *i *with residue *j*. In the work of Schlosshauer and Olsson [13], the measure of validity for matching residue *i *with residue *j *was based on substituting the discrete function "max" in the dynamic programming algorithm with a parameter-dependent analog function. It allowed evaluating possible suboptimal alternatives for the chosen aligned pair of residues, thus also allowing a numerical evaluation of the accuracy of their matching. This numerical index calculated for each pair of residues serves as a measure of the local accuracy of the alignment.

As opposed to the works mentioned above, our evaluation of algorithmic alignment methods was based not on the assessment of the alignment results for a few selected positions, but on the comparison of algorithmic alignments with the GS alignment as a whole over the total length of the sequences (see [14-16]). From the results of comparison of structural alignments with local algorithmic Smith-Waterman alignments Sunyaev *et al*. [16] made a conclusion that the possibility of reconstruction of structural alignments from algorithmic ones depends on the degree of similarity of appropriate proteins; besides, examination of internal structures of both alignments allowed to develop a more efficient procedure for aligning two sequences, taking into account not only the mean level of their identity, but also the distribution of more or less similar regions in the sequences in the structural alignment.

However, all the works cited above had a common fault: algorithmic alignments were compared not with the true evolutionary alignments (which were unknown!), but with their approximations. This introduced an error in the results, which could not be estimated by the usual direct methods. We suggest using a comparison of artificially generated sequences to evaluate the quality of alignment algorithms, because the GS alignment for such sequences is known from the very beginning. A similar numerical experiment was described in [17,18]. However, generation of the test set of sequences in [17] did not reflect completely available data on the evolutionary process, because insertions and deletions were generated in accordance with an over-simplified algorithm. In the work [18], to generate a set of test sequences, a different evolution model was used, which had been described in [19-21]. The model included both point mutations and indels. Numerical values of the mean accuracy were obtained for the global alignment algorithm with affine gap penalties (the global version of the Smith-Waterman algorithm) for various evolutionary distances.

The purpose of our work was to determine conditions of preferred applicability of the local and global versions of the algorithm for determination of optimal alignments with an affine penalty function for indels [22]. Thereinafter, for the sake of brevity, this algorithm will be called the "Smith-Waterman algorithm". As is well known, the global algorithm finds such positions for gaps in the sequences, which correspond to the maximum value of the difference between summed weights of matched residues and summed penalties for the gaps. A local algorithm allows finding the optimal alignment of two fragments of the studied sequences, whereby the regions before and after the fragments forming the alignment with the maximum weight are not taken into account when the weight is calculated. Thus, unlike the global algorithm, the local algorithm allows determining not only optimal positions for gaps in some fragments, but also the fragments themselves, which provide for their appropriate positioning.

Our task was to determine the relative quality of alignments obtained through the global and local algorithms versus the degree of homology of similar regions in the sequences (the "cores") and the length of nonhomologous regions at the ends of the sequences (the "consoles"). In particular, we tried to determine the application threshold for the global algorithm, i.e. the values of the above-mentioned parameters, which provided for the same or better quality of alignments by using the global algorithm in comparison to the quality of alignments by using the local algorithm (see the definition of the alignment quality in section 2.3).

## 2. Methods

### 2.1 Preparation of test sets of sequence pairs

#### 2.1.1 General description of sets

To carry out computer experiments we prepared 224 test sets, each set including 1000 sequence pairs ("test pairs"). All sets were prepared using the same technique, and different sets have different technique parameters (see below).

A test sequence pair consists of two sequences generated independent of each other from a common initial sequence ("ancestor"), each of their compared sequences consisting of a homologous part ("core") and nonhomologous parts ("consoles") surrounding the core.

The generation of test pairs is described in the next subsection. It has the following parameters:

1) PAM is the measure of evolutional distance from the common ancestor to the sequence cores (see [19]);

2) *r *is the ratio of the total length of consoles to the length of the common ancestor of sequence cores (this ratio is accepted to be equal for the both sequences of a pair and, as a result, the total lengths of consoles are equal for the both sequences);

3) *c *is the ratio of the absolute value of the difference between console lengths to their total length (this ratio is the same for the both sequences, but for sequence *S1 *the left console is no longer than the right one, and vice versa for sequence *S2*).

To generate test sets with such parameters we used the following values:

1) *PAM = *30, 60, 120, 240 PAM;

2) *r = *10%, 20%, 50%, 100% , 200%;

3) *c = 0% *(the consoles are of the same length), *10%, 20%, ... 100% *(one console is missing).

**Figure 1:**
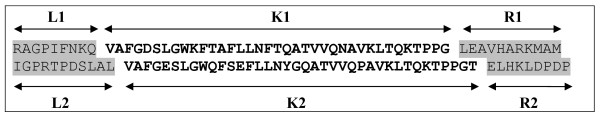
**Modified sequences with consoles**. Here **L1, L2, K1, K2, R1**, **R2 **are left consoles, cores and right consoles of the first and second modified sequences; their lengths |**L1**| = |**R2**| = 9, |**L2**| **= **|**R1**| = 11, |**K1**| ≠ |**K2**| (inequality is explained by differing length of random insertions and deletions). Then *r *= (|**L1**| + |**R1**|)/|**P**| = (9+11)/200 = 0.1, ***c ***= (|**R1**| - |**L1**|)/|(|**L1**| + |**R1**|) = 2/20 = 0.1, where |**P**| = 200 is the length of the initial sequence.

Test sets were prepared for all possible combinations of the above parameters, i.e. in total 224 test sets were prepared (including sequence sets without consoles); the meaning of the *r *and *c *values is explained in Figure 1.

#### 2.1.2 Description of generation of a test sequence pair

The process of generation of a test sequence pair consists of the following stages:

(i) Generation of a common ancestor *P *of cores of test sequences;

(ii) Generation of cores *K1 *and *K2 *of test sequences in accord with the *PAM *value;

(iii) Estimation of the total length of consoles for each of the sequences in accord with the *r *values;

(iv) Estimation of the length of each console in accord with the *c *value;

(v) Generation of consoles *L1, R1 *(the left and right consoles of the first sequence), *L2, R2 *(the left and right consoles of the second sequence);

(vi) Construction of desired test sequences *S1, S2:*

S1=L1∙K1∙R1;S2=L1∙K1∙R1.

To solve this task we used the following evolution model, which is a version of the evolution model used in our previous paper [18]. Therein we analyzed the alignment of the ancestor sequence *S0 *and generated sequence *S1 *from it, whereas here we examine alignments of sequences *S1 *and *S2 *generated from the common ancestor sequence *S0*.

The ancestor sequence is generated as a random Bernoulli amino acid sequence of about 200 a.a., probabilities of selection of amino acids were taken as advised in [19]. Consoles are generated in the same way (independent of each other and of the ancestor sequence). Their lengths are estimated using the formulas (here and lower *|w| *is the length of a symbolic sequence *w*, i.e. *|P| *is the length of sequence *P *which is a common ancestor for cores of compared sequences):

$$\left|L1\right|=\left|P\right|∙r∙\left(1-c\right)∕2;\;\;\left|R1\right|=\left|P\right|∙r∙\left(1+c\right)∕2;$$

$$\left|L2\right|=\left|P\right|∙r∙\left(1+c\right)∕2;\;\;\left|R2\right|=\left|P\right|∙r∙\left(1-c\right)∕2.$$

Generation of cores is described in subsection 2.1.3.

#### 2.1.3 Construction of homologous regions of test sequences (cores)

Cores *K1 *and *K2 *were constructed of an ancestor sequence *P *independently and according to the same procedure. The procedure consisted of two stages.

At the first stage, insertions and deletions were incorporated into the ancestor sequence. Let us say that there is an indel at position *i *(*i *from *1 *to *L+1*) if there is an insertion before position *i *or a deletion beginning from this position *i*; position *L+1 *corresponds to the insertion at the sequence terminus. The probability of an indel at a given position was calculated by the formula:

(1) $$P\left(indel\right)=0.0224-0.0219\cdot e^{\left(-0.01168*PAM\right)},$$

where PAM is the number characterizing the evolutional distance between the original and modified sequences [19].

For each position *i, i = 1, ..., L+1*, we determined by a random choice if there is an indel. Then we decided at random if an insertion or deletion appeared, the probability of the deletion was chosen to be 0.55. This value was determined empirically, provided that the total length of insertions and deletions was equal. When p(ins) = p(del) = 0.5 at the accepted procedure, the total length of insertions was greater than that of deletions. The length of an insertion or deletion was chosen at random from Zipfian distribution which, as stated in [20], is independent of the evolutional distance. If the chosen deletion length exceeded the distance from position *i *to the sequence terminus (in particular, at *i = L+1*) or the beginning of the deletion coincided with the already deleted position, the attempt was ignored. Ignored was also the insertion if its beginning coincided with the elongation of the earlier made insertion or deletion. Thus, we prevented insertions and deletions, whose length was not equal to the value obtained from the preset distribution.

At the second stage, point mutations were inserted in the obtained sequence. At this, mutations were naturally inserted only in the remaining sites of the original sequence. A cycle of inserting mutations consisted in the following. A substitution is made in every position with certain probability, the probability of origin of a new symbol in this position being determined by the probability matrix *PAM1 *[19]. This cycle changes approximately so many times as is the value of *PAM*. Table 1 shows average portions of coincidence of the ancestor and mutant sequences (%id) and parameter *PAM*.

**Table 1:** Correspondence of *%id *and *PAM*.

%id	PAM	%id	PAM
99	1	50	80
95	5	45	94
90	11	40	112
85	17	35	133
80	23	30	159
75	30	25	195
70	38	20	246
65	47	15	328
60	56	10	489
55	67	5	830

The average fraction of coincidence of the ancestor and mutant sequences (%id) and the corresponding *PAM *value. Only substitutions are allowed in the mutant sequence.

The letter composition of insertions was generated analogous to original sequences. It should be noted that contrary to the scheme used in [18], in this case the total length of insertions-deletions was not a constant value for all pairs of sequences from one test set and obeyed the law of random numbers (which is closer to the real evolutional set).

### 2.2. Comparison of "ancestor-descendant" and "descendant1-descendant2" tests

In the above procedure, test pairs of sequences are generated according to the "descendant1-descendant2" scheme (i.e. two descendants of a common ancestor are compared) rather than by the "ancestor-descendant" scheme (the ancestor sequence is compared to its descendant). The first scheme in a better way models a comparison of real sequences. However the *PAM *parameter, used traditionally for characterizing the evolutional distance, is second-scheme-oriented, but since the parameter is universal, nothing prevents its use for estimating the distance between descendants of a common ancestor.

Table 2 shows how different values of *PAM *correspond to the ratio of the test sequences generated according to the "descendant1-descendant2" scheme. The table has two data blocks ("ancestor-descendant" and "descendant1-descendant2"). The first block is of two columns each characterizing reference alignment (see subsection 2.3) of the ancestor sequence and the core generated from it in the course of the process described in subsection 2.1. Column "%id" lists average percentage of column-coincidences among all columns describing letter comparison. Column "%indel" describes the percentage of column-indels among columns of reference alignment. The "descendant1-descendant2" block has four columns. Columns "%id" and "%indel" have the same meaning as in the "ancestor-descendant" block, though they denote reference alignment of two descendants of a common ancestor. Columns *PAM(%id)*and *PAM(%indel) *list *PAM *values at which corresponding characteristics could be obtained for "ancestor-descendant" pairs. The table shows that from the point of view of *%id*, the use of a diverging scheme is equivalent to the use of a sequential scheme at a twice higher *PAM *value. Nonetheless the number of indels for the "descendant1-descendant2" scheme with parameter *PAM *is essentially higher than for the "ancestor-descendant" scheme with parameter 2·*PAM*.

**Table 2:** Characteristics of modified sequences generated according to the sequential ("ancestor-descendant") and diverging ("descendant1-descendant2") schemes.

PAM	"Ancestor-descendant"		"Descendant1-descendant2"			
	
	%Id	%Indel	%id	PAM (id)	%indel	PAM (indel)
30	74.9%	3.3%	57.9%	60.55	6.6%	68.56

60	57.9%	5.4%	37.6%	122.25	10.7%	202.14

120	37.6%	7.5%	20.1%	245.08	14.7%	>830

240	20.2%	8.9%	10.5%	473.87	17.3%	> 830

Lines correspond to different values of *PAM*. The figures in columns are described in the text (subsection 2.2). All data are given as selected from 1000 sequence pairs. For control, the same data were obtained for a variety of 10,000 pairs; the results do not differ from those given in the table with an accuracy of tenths of a percent.

### 2.3 Alignment of sequences with consoles

Alignment of generated sequences was performed using two variations of the Smith-Waterman algorithm - the global and local ones. For global alignment the substitution weight matrix PAM250 and gap-open and gap-extension penalties 14 and 2 were used; for local alignment the Gonnet250 matrix and penalties 10 and 0.5 were used. Such parameter values were chosen on the base of the preliminary computer experiments [15,17] which allowed getting the best values of the quality of alignments (see subsection 2.4) through all test sets. These values are close to those commonly used.

Prior to the tests with sequences of variable homology along the sequence, we investigated the dependence of the quality of the alignment on the applied substitution matrix and gap-open and gap-extension penalties. For this purpose the following numerical experiment was held.

Test sets including 1000 pairs of sequences each were generated according to the "sequential" scheme of evolution with average distances between ancestors and descendants of 60, 100, 200, 300 PAM (see the description of "sequential" and "diverging" schemes in item 2.2).

Pairs of sequences of each test set were aligned by global version of Smith-Waterman algorithm using two matrices: "native" (i.e. the PAM matrix corresponding to the evolutionary distance between sequences) and PAM250. Values of the measures of similarity (2.4) of algorithmic alignments via the reference alignments derived from "native" matrix and the PAM250 matrix, are practically the same (difference less than 1%, see the table in additional file 1, sheet **PAM250**). As we observed, the high homologous proteins (id > 45%) can be successfully aligned, using a matrices designed for large evolutionary distances. At the same time, attempts to align low homologous proteins, using a matrix designed for shorter distances, significantly deteriorate the results, signifying mismatch of algorithmic and reference alignments.

This is confirmed by a more complete numerical experiment. For this aim, each pair of sequences of all test sets were aligned using matrices PAM60, PAM100, PAM200, and PAM300. Gap-open and gap-extension penalties ranged from 10 to 20 and from 1 to 5, respectively (see the data in additional file 1, sheet **Max Values**). A practical conclusion of this finding, obviously, is that if the evolutionary distance between the sequences is unknown, the evolutionary distance corresponding to applied matrix, should be certainly greater than expected distance between the sequences.

Complete data on the comparative analysis of weight matrices are given in additional file 1, sheet **All Values**.

### 2.4 Determination of the quality of algorithmic alignments

**Figure 2:**
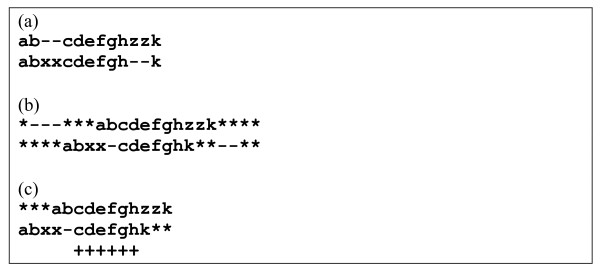
**Determination of the quality of alignment of sequences with consoles**. (a) Reference alignment of sequence cores. (b) Algorithmic alignment of sequences. (c) Fragment of algorithmic alignment the quality of which should be assessed ('+' means that comparison is present in the reference alignment).

We are fascinated how close the alignments obtained by the program are to the reference alignments, i.e. alignments with aligned positions generated from the same position of the ancestor sequence. To assess the closeness ("the alignment quality"), we have used Accuracy and Confidence measures described elsewhere [14-16]. It should be underlined that in the reference alignment the console positions were accepted to be unaligned. Therefore when calculating the number of comparisons in the algorithmic alignment, we took only columns in which at least one symbol would belong to a homologous region. To explain the above, we will analyze the following example. Let K1 = **"abcdefghzzk" **and K2 = **"abxxcdefghk" **be cores of the compared sequences (their reference alignment is given in Figure 2a). Then let S1 = "****abcdefghzzk****" and S2 = "****abxxcdefghk****" be sequences with consoles (asterisks designate console symbols that are insignificant for us and probably diverse). The algorithmic alignment of sequences with consoles is shown in Figure 2b, and its fragment whose quality is to be assessed is given in Figure 2c. We get the following.

Number of columns without deletions in the reference alignment:

G=9;

Number of columns without deletions in the algorithmic alignment:

A=13;

Number of common columns without deletions:

I=6;

Accuracy:

Accuracy=I∕G=6∕9;

Confidence:

Confidence=I∕A=6∕13.

## 3. Results and Discussion

We have analyzed the dependence of the quality (i.e. accuracy and confidence) of the global and local alignments of console sequences versus the following values: (1) the evolutional distance between homologous fragments of sequences ("cores"); (2) the console length; and (3) console asymmetry ("shifted cores").

### 3.1. Symmetrical consoles

Table 3 shows the dependence of the accuracy and confidence values at a symmetrical position of the core (*c = 0*) and at all *PAM *and *r *values analyzed (see subsection 2.1.1). It is seen that when the evolutional distance *PAM *and the console length *r *grow, the accuracy and confidence values decrease. In this case, with the same evolutional distance and console length, the accuracy and confidence values of the global alignment are somewhat higher than those of the local alignment, this difference increasing with the growth of the evolutional distance. For example, at an evolutional distance of 240 PAM the accuracy of the global alignment is by 18.6% higher than that of the local alignment, whereas the confidence is only 16.2% higher.

**Table 3:** Accuracy and confidence of global and local alignment at symmetrical consoles

Evolutional distance, PAM	Console length, %	Global alignment		Local alignment	
		
		Accuracy, %	Confidence, %	Accuracy, %	Confidence,%
30	0	98.98	98.86	98.19	98.57

30	10	98.90	98.62	98.23	98.20

30	20	98.77	98.38	98.11	97.95

30	50	98.50	97.88	97.91	97.45

30	100	98.47	97.82	97.91	97.36

30	200	98.40	97.61	97.65	96.93

60	0	95.88	95.42	93.19	94.37

60	10	95.89	95.25	93.61	93.91

60	20	95.24	94.38	93.29	93.20

60	50	94.59	93.22	92.92	91.99

60	100	94.65	93.05	92.84	91.46

60	200	94.35	92.77	92.57	91.21

120	0	82.47	81.47	71.76	76.71

120	10	81.13	79.79	71.34	74.16

120	20	79.30	77.59	71.16	72.64

120	50	77.95	75.77	70.51	69.77

120	100	76.70	74.11	69.48	67.78

120	200	76.32	73.34	68.69	66.79

240	0	38.86	38.31	15.14	19.30

240	10	36.80	35.90	15.50	18.12

240	20	33.06	32.05	14.22	15.71

240	50	30.71	29.46	14.22	14.49

240	100	27.93	26.63	12.79	12.43

240	200	25.33	23.90	9.27	8.88

The dependence of Accuracy and Confidence values on the evolutional distance between cores (Evolutional distance, PAM) and relation of the total length of consoles to the core length (Console length, %) is shown. Of notice is the qualitative predominance of the global algorithm at the evolutional distance of 240 PAM.

Thus, at a symmetrical position of the consoles, the global algorithm has higher resistance to the increase of evolutional distance and console length than the local algorithm.

### 3.2. Asymmetrical consoles

**Figure 3:**
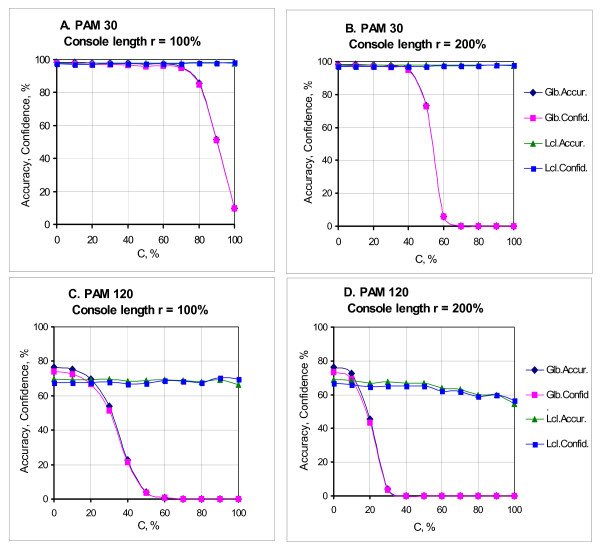
**Dependence of accuracy and confidence of alignments versus the core shift *c***. A, B - evolutional distance of 30PAM; C, D - evolutional distance of 120PAM. A, C - console length r = 100%; B, D - console length r = 200%. The accuracy and confidence of the global (*Glb.Accur., Glb.Conf*.) and local (*Lcl. Accur., Lcl.Conf*.) alignments at the console length making 100% and 200% of the length of cores and at evolutional distances between the cores 30PAM and 120PAM. The parameter *c *(see its definition in subsection 2.1.1) determines the core shift value. The plots for accuracy and confidence are compatible at evolutional distance of 30PAM.

In contrast, if homologous regions of aligned sequences are shifted in opposite directions from the center of sequences, both the accuracy and confidence of the global alignment decrease remarkably (see Figure 3). In such a case, at a fixed evolutional distance between the sequence cores and the total length of consoles, degradation of the global alignment becomes sharp in a relatively narrow range of changing the console asymmetry *c *(see the definition of *c *in subsection 2.1.1). So, at the evolutional distance of 30PAM, the total length of consoles 100% of the core length and the increase of console asymmetry from 80% to 90%, the accuracy and confidence drop from 85% to 51%. At the total length of consoles 200% and an increase of console asymmetry from 50% to 60%, a much sharper decrease in the accuracy and confidence takes place (from 73% to 6%).

It should be noted that the above accuracy values of global alignments are much lower than the values given in previous papers (see, for example, [18]). This is explained by the fact that we analyze the sequences with consoles, whereas in the cited and some other papers only alignments of sequences homologous as a whole were considered.

When the evolutional distance between homologous regions of sequences continues to increase, this tendency becomes more pronounced. Thus, when the evolutional distance is 60 PAM, the total console length is 100% and the core shift changes from 50% to 70%, the accuracy value decreases from 87% to 30%, and the confidence value decreases from 85% to 29%. When the evolutional distance is 120 PAM, the total console length is 50% and the core shift changes from 40% to 60%, the accuracy value decreases from 67% to 38%, and the confidence value decreases from 64% to 36%. When the evolutional distance is 240 PAM and the console length is 50%, the accuracy and confidence values begin to decrease at a shift of 10%.

So, an increase in the evolutional distance between homologous regions of sequences causes a noticeable decrease in the quality of global alignment, a considerable decrease of the quality of global alignment taking place at a decreasing length of consoles and a diminishing shift of the core from the center of sequences.

The entire records of the dependence of accuracy and confidence values on the three parameters are given in Additional File 2.

It is essential that at all considered values of PAM, length and asymmetry of consoles, there is no remarkable decrease in the quality of local alignment. In other words, in case of asymmetrical consoles, the local algorithm is more resistant to the increase in evolutional distance and console length than the global algorithm.

Thus, we can conclude that there exists some *threshold value *of the three parameters: extent of core homology, total length of consoles and asymmetry of consoles. Prior to this value, the quality of the global alignment is higher than that of the local alignment, but above this value the global alignment quality decreases sharply, whereas the local alignment quality remains the same.

### 3.3. Determination of the "slope zone"

The plots in Figure 3 show that when the asymmetry of consoles *c *changes from 0 to 100% (at fixed evolutional distances between cores and the total length of consoles), the accuracy and confidence of global alignments decrease. In this case the decrease is sharp in a relatively narrow (~ 20%) range of *c *values; this range will be called the slope zone. In this section we will demonstrate how to predict theoretically where this area is. It will be accepted that the substitution weight matrix and gap penalties are fixed (see subsection 2.3).

Let *P *be a pair of test sequences, *Lker(P) *be the average length of cores of these sequences, *Score (P) *be the weight of reference alignment of cores for the considered set. The *density *of cores alignment for pair *P *will be the relation:

$$D_{ker}\left(P\right)=Score\left(P\right)∕L_{ker}\left(P\right).$$

Let us consider one of the sets of test sequences described in subsection 2.1. Using *D_ker _*and *L_ker _*we will denote mean values: *Dker(P) *and *Lker(P)*, respectively, for the sequences in the set under discussion.

Then let *Lcon = r·Lker *be the length of consoles, *Dcon *be the total average relation ofalignment weights of two independent random sequences of equal length to the sequence length, *c *be the asymmetry of consoles (see the designations in subsection 2.1). Then the mean weight *Scoreglob *of the reference alignment of a pair of test sequences generated by the scheme given in subsection 2.1 with the pairs *Lker*, *PAM, r*, and *c *is determined by the following equation:

(2) $$Score_{glob}=L_{ker}\cdot D_{ker}+L_{con}\cdot D_{con}\cdot \left(1-c\right)-2\cdot GEP\cdot L_{con}\cdot c-2\cdot GOP,$$

where *GOP *and *GEP *are gap-open and gap-extension penalties.

By fixing all members in equation (2), except the asymmetry values *c*, we will get an equation for the dependence of the mean weight of reference alignments *Scoreglob *versus the asymmetry of sequences *c*. Let us analyze the relation

$$D_{glob}\left(c\right)=Score_{glob}\left(c\right)∕\left(L_{ker}+L_{con}\right)$$

of the mean weight of reference alignments to the mean length of test sequences in a corresponding set. The *D_glob _*value will be called the *mean density *of reference alignments. Similarly, the mean density of random alignments *D_rand _*will be the relation of the mean weight of optimal alignment of two independent random sequences to the length of these sequences (see the details in subsection 3.4).

**Figure 4:**
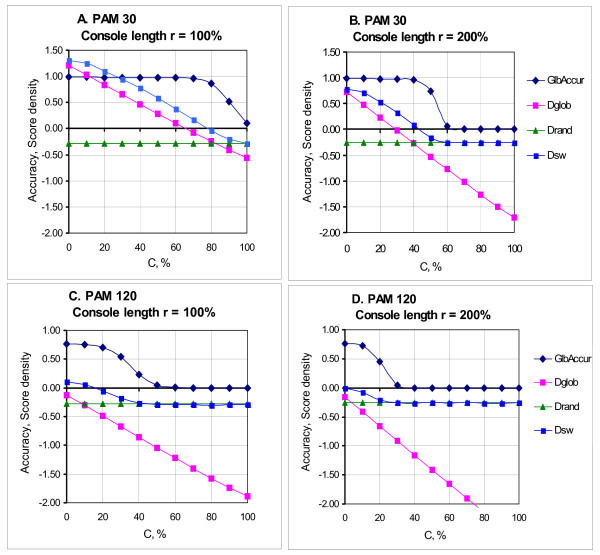
**"Slope zone" of the Smith-Waterman global alignment and density of reference and random alignments**. A, B - evolutional distance of 30PAM; C, D - evolutional distance of 120PAM. A, C - console length r = 100%; B, D - console length r = 200%. Mean densities of reference (*D_glob_*) and random (*D_rand_*) alignments and mean accuracy of Smith-Waterman global alignments (*Glb.Accur*) versus the asymmetry value of consoles *c *at evolutional distances 30 and 120PAM and consoles of 100% and 200% length of the core length of the sequences. The intersection of straight lines - plots of the *D_glob _*and *D_rand _*values corresponds to the beginning of the "slope zone" of the accuracy plot (see also Fig. 3). Densities of Smith-Waterman global alignments are given for comparison. Here the intersection of plots for *D_glob _*and *D_rand _*values is typically close to the point where the plot for *D_sw _*passes the zero value. At rather high *c *values the density of algorithmic global alignments (*D_sw_*) is close to the density of random alignments (*D_rand_*).

Computer experiments (see Figure 4) showed that a sharp decrease in the accuracy and confidence of global alignments takes place close to the asymmetry value *c *which is the root of equation

(3) $$D_{glob}\left(c\right)=D_{rand}$$

(here the length of random sequences is *L_ker_+L_con_*). In other words, it is impossible to restore the reference alignment if its weight does not (almost) differ from the alignment weight of random sequences. Using the *L_ker_*, *PAM, r *values, the above stated allows us to roughly determine the range of asymmetry values *c *at which the quality of global alignment decreases.

### 3.4. Determination of density of random alignments

**Figure 5:**
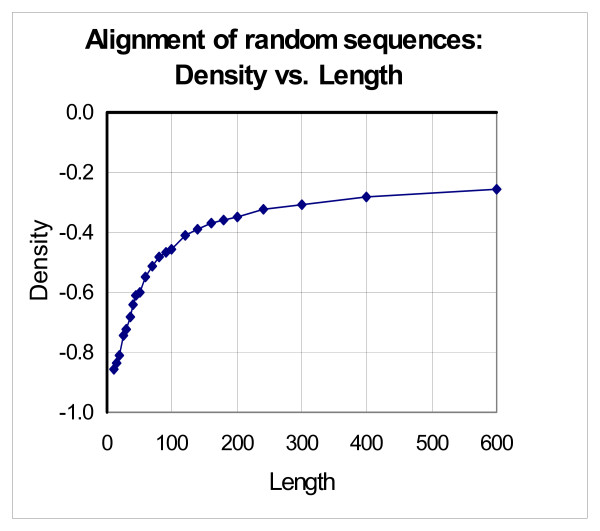
**Dependence of density of global alignment of random sequences versus their length**. Pairs of random sequences from 10 to 600 a.a. were obtained according to the procedure described in Methods (subsection 2.1.2). The pairs were aligned using the global version of Smith-Waterman algorithm (substitution weight matrix PAM250, gap-open and gap-extension penalties GOP = 14, GEP = 2).

To find the *D_rand _*value, we determined the weight dependence of two random sequences versus their length (see Figure 5). Pairs of random sequences from 10 to 600 a.a. were obtained according to the procedure described in subsection 2.1.2. The pairs were aligned using the global version of Smith-Waterman algorithm with the substitution weight matrix PAM250 and gap-open and gap-extension penalties GOP = 14, GEP = 2.

As seen from Figure 5, the lowest density corresponds to the shortest sequences of 10 a.a. and is -0.858. Let us mention for comparison that for the substitution weight matrix PAM250 and the amino acid frequency distribution from [19] the mean weight of symbol comparison is -0.863, which is estimated by the formula:

$$\sum_{i=1..n}P_{i}\;\sum_{j=1..n}P_{j}\;S_{ij},$$

where *Pi *is the probability of appearance of the *i*-th amino acid residue and *S_ij _*is the substitution weight.

### 3.5. Comparison with other algorithms

Additionally, we compared three algorithms: Needleman-Wunsch [1], Smith-Waterman [2] and GAP3 [5]. A generalized global alignment algorithm (GAP3) is a development of the standard Needleman-Wunsch dynamic programming algorithm designed for comparing sequences with intermittent similarities, an ordered list of similar regions separated by different regions. (In difference to the algorithm given in [1], the algorithm described in [5] besides the usual weight substitution matrix and gap-open and gap-extension penalties, requires an additional parameter - constant penalty for each difference block.)

A comparison test was carried out on thirteen sets of 1,000 pairs of sequences each with the following parameters (2.1.1): 1) evolutionary distance from a common ancestor to the sequence cores *PAM *= 120 PAM; 2) the ratio of the total length of consoles to the length of the common ancestor of sequence cores *r *= 20, 50, 100%; 3) the ratio of the absolute value of the difference between console lengths to their total length *c *= 0, 30, 50, 70%.

We used the GAP3 program available online (http://deepc2.psi.iastate.edu/aat/gap3/), with matrix PAM250, and gap-open and gap-extension penalties equal to 14 and 2 and constant penalty for each difference block equal to 40. The results of comparison are shown in Table 4.

**Table 4:** Accuracy and confidence of global, local and GAP3 alignment at various consoles

PAM	Console length r, %	Core shift c, %	Global alignment		Local alignment		GAP3	
			
			**Accur**., %	**Confid**., %	**Accur**., %	**Confid**., %	**Accur**., %	**Confid**., %
120	0	0	82.47	81.47	71.76	76.71	71.42	86.60

120	20	0	79.30	77.59	71.16	72.64	71.58	84.98

120	20	30	75.64	73.39	71.37	72.99	72.26	85.02

120	20	50	72.23	69.83	70.17	72.18	72.19	84.78

120	20	70	69.47	66.93	70.81	72.94	71.03	84.80

120	50	0	77.95	75.77	70.51	69.77	72.66	84.00

120	50	30	72.04	69.08	71.47	70.91	73.06	84.92

120	50	50	53.53	50.96	69.34	69.36	71.80	83.62

120	50	70	20.18	18.98	70.37	71.03	72.14	84.16

120	100	0	76.70	74.11	69.48	67.78	72.18	83.39

120	100	30	53.69	51.45	69.63	68.09	72.12	83.25

120	100	50	4.15	3.71	68.66	67.23	72.15	83.84

120	100	70	0.08	0.05	68.95	68.47	72.47	83.04

The dependence of Accuracy and Confidence values of tree types of alignment on the evolutional distance between cores (PAM), relation of the total length of consoles to the core length (Console length r, %) and the ratio of the absolute value of the difference between console lengths to their total length (Core shift c, %) are shown.

The table shows that in comparison with Smith-Waterman local alignment, in all cases the GAP3 alignment benefits slightly in Accuracy, but advantage in Confidence is significantly larger (it means that GAP3 makes a little more correct matches than the first algorithm, making fewer false matches).

As for comparison with global Needleman-Wunsch alignments, in the case without consoles the GAP3 alignments have a much lower Accuracy and a slightly higher Confidence. For symmetric consoles simultaneously with increase in their length (Core shift = 0, Console length = 20,...,100%), this tendency is reduced and becomes less prominent. It was mentioned above that as a result of the displacement of the cores of aligned sequences in the opposite direction from the center, the reliability of the global alignment is markedly reduced. However, a local alignment has no such a tendency. As shown, at the evolutionary distance of 120PAM, the GAP3 alignment is not only resistant to displacement, but also resistant to increasing the length of nonhomologous consoles.

## 4. Conclusion

The study has revealed regularities allowing for defining more exactly the areas of effective application of every algorithm: when consoles are positioned symmetrically, the global algorithm is more resistant to increasing evolutional distance and console length than the local algorithm (about 10% accuracy and about 8% confidence at 120PAM and up to 20% accuracy and confidence at 240PAM); quite the opposite, when consoles are asymmetrical, the local algorithm is more resistant to increasing evolutional distance and console length than the global algorithm. The boundary of the global algorithm preference is determined roughly by the value of asymmetrical position of homologous fragments of sequences (cores) at which the reference alignment density is almost equal to the density of random sequence alignment. The mean divergence of 5 ÷ 10%, which is typical both of accuracy and confidence of global and local alignments at a symmetrical position of cores, preconditions the developing of a combined method for making a more reliable alignment.

## Competing interests

The authors declare that they have no competing interests.

## Authors' contributions

VOP performed all computations, participated in planning the research and wrote the manuscript. MAR and VGT designed the study and contributed to the manuscript. All authors fulfilled the analysis of the results, read and approved the final manuscript.

## Supplementary Material

Additional File 1 **Substitution matrix and gap-open and gap-extension penalties test for optimal global alignment**. Accuracy and Confidence values for global alignments versus the following: (1) evolutional distance between sequences (*PAM*); (2) substitution matrix; (3) gap open and gap extension penalties (*GOP, GEP*). Click here for file

Additional File 2 **Dependence of accuracy and confidence values on three parameters (*PAM*, *r*, and *c*)**. Accuracy and Confidence values for global and local alignments versus the following: (1) evolutional distance between homologous fragments of sequences (*PAM*); (2) console length (*r*); (3) console asymmetry (core shift, *c*). Click here for file
